# Time‐of‐day dependent effects of contractile activity on the phase of the skeletal muscle clock

**DOI:** 10.1113/JP279779

**Published:** 2020-07-01

**Authors:** Denise Kemler, Christopher A. Wolff, Karyn A. Esser

**Affiliations:** ^1^ Department of Physiology and Functional Genomics University of Florida 1345 Center Drive Gainesville FL 32610 USA; ^2^ Myology Institute University of Florida 1200 Newell Drive Gainesville FL 32610 USA

**Keywords:** circadian phase, electrical pulse stimulation, exercise, skeletal muscle

## Abstract

**Key points:**

- Disruptions in circadian rhythms across an organism are associated with negative health outcomes, such as cardiometabolic and neurodegenerative diseases.
- Exercise has been proposed as a time cue for the circadian clock in rodents and humans.
- In this study, we assessed the effect of a single bout of endurance exercise on the skeletal muscle clock *in vivo* and a bout of muscle contractions *in vitro*.
- Timing of exercise or contractions influences the directional response of the muscle clock phase *in vivo* and *in vitro*.
- Our findings demonstrate that muscle contractions, as a component of exercise, can directly modulate the expression of muscle clock components in a time‐of‐day dependent manner.

**Abstract:**

Exercise has been proposed to be a zeitgeber for the muscle circadian clock mechanism. However, this is not well defined and it is unknown if exercise timing induces directional shifts of the muscle clock. Our purpose herein was to assess the effect of one bout of treadmill exercise on skeletal muscle clock phase changes. We subjected PERIOD2::LUCIFERASE mice (*n* = 30F) to one 60 min treadmill exercise bout at three times of day. Exercise at ZT5, 5 h after lights on, induced a phase advance (100.2 ± 25.8 min; *P* = 0.0002), whereas exercise at ZT11, 1 h before lights off, induced a phase delay (62.1 ± 21.1 min; *P* = 0.0003). Exercise at ZT17, middle of the dark phase, did not alter the muscle clock phase. Exercise induces diverse systemic changes so we developed an *in vitro* model system to examine the effects of contractile activity on muscle clock phase. Contractions applied at peak or trough *Bmal1* expression induced significant phase delays (applied at peak: 27.2 ± 10.2 min; *P* = 0.0017; applied at trough: 64.6 ± 6.5 min, *P* < 0.0001). Contractions applied during the transition from peak to trough *Bmal1* expression induced a phase advance (49.8 ± 23.1 min; *P* = 0.0051). Lastly, contractions at different times of day resulted in differential changes of core clock gene expression, demonstrating an exercise and clock interaction, providing insight into potential mechanisms of exercise‐induced phase shifts. These data demonstrate that muscle contractions, as part of exercise, are sufficient to shift the muscle circadian clock phase, likely through changes in core clock gene expression. Additionally, our findings that exercise induces directional muscle clock phase changes confirms that exercise is a bona fide environmental time cue for skeletal muscle.

## Key points

- Disruptions in circadian rhythms across an organism are associated with negative health outcomes, such as cardiometabolic and neurodegenerative diseases.
- Exercise has been proposed as a time cue for the circadian clock in rodents and humans.
- In this study, we assessed the effect of a single bout of endurance exercise on the skeletal muscle clock *in vivo* and a bout of muscle contractions *in vitro*.
- Timing of exercise or contractions influences the directional response of the muscle clock phase *in vivo* and *in vitro*.
- Our findings demonstrate that muscle contractions, as a component of exercise, can directly modulate the expression of muscle clock components in a time‐of‐day dependent manner.

## Introduction

The circadian clock is an evolutionarily conserved regulatory mechanism that allows organisms to adapt, respond and entrain to their environment. By integrating/responding to environmental signals (i.e. time cues), the circadian clock coordinates daily oscillations of behaviour, metabolism and gene expression at the organismal and tissue‐specific levels driving circadian rhythms (Golombek & Rosenstein, 2010). Contained in virtually all cells throughout the body, the mammalian circadian clock is an endogenous self‐sustaining transcription–translation feedback loop that directs a daily programme of gene expression. Consistent across tissues, the core clock transcription factors *Bmal1* and *Clock* drive the expression of *Period1/2* and *Cryptochrome 1/2* which in turn repress their own expression by inhibiting *Bmal1* and *Clock* transcriptional activity (Ko & Takahashi, 2006; McCarthy *et al*. 2007; Mohawk *et al*. 2012; Partch *et al*. 2014). The circadian cycle takes ≈24 h to be completed and thus defines the period length (e.g. interval between two peaks) of the circadian clock output.

While the period length is fixed, the circadian phase is variable and represents a temporal reference point relative to a fixed event. The most prominent example is the central clock in the brain (suprachiasmatic nuclei, SCN) where the circadian phase of a rest/activity pattern is determined by the naturally occurring light/dark cycle (fixed event) driving most circadian behaviour (Merrow *et al*. 2005; Wright *et al*. 2013). Environmental time cues that are capable of entrainment (i.e. setting the circadian phase) are called zeitgebers and while light is the most prominent time cue, it primarily entrains the SCN central clock. However, additional non‐photic zeitgebers exist, such as feeding, activity and stress, all of which serve as time cues to peripheral tissue clocks (Fuller *et al*. 2008; Mistlberger & Antle, 2011; Sujino *et al*. 2012; Tahara & Shibata, 2018). Therefore, light acts as a zeitgeber for the central clock, setting a phase for all clocks throughout the body. However, non‐photic time cues can act on peripheral tissues, influencing the phase of peripheral tissue clocks with the potential for putting them out of alignment with the phase set by the SCN (Maywood & Mrosovsky, 2001; Wolff & Esser, 2012; van der Vinne *et al*. 2018; Koronowski *et al*. 2019).

Time cues must meet certain criteria to qualify as a true zeitgeber. Specifically, an environmental time cue must interact with the core molecular clock mechanism to shift the phase of the endogenous rhythm. This shift in phase can be a negative (delay) or positive (advance) and its direction is dependent upon when the environmental cue was applied. Finally, the phase shift must be persistent, in contrast to a local reset in which case the phase returns to its old status before the time cue was applied. A powerful tool to investigate the effects of zeitgeber on the circadian phase is the phase response curve (PRC). Phase response curves are a graphic representation of the dynamics (phase shift direction and magnitude) of a circadian oscillator when stimulated by a zeitgeber at different times of day (reviewed in Johnson, 1999). When a PRC shows a phase shift during the subjective night (usually a delay in the first half and an advance in the second) they are called ‘photic PRC’ (Daan & Pittendrigh, 1976; Rusak & Boulos, 1981). Those curves with phases that advance during the dark phase (are so‐called ‘non‐photic’ PRC and happen in response to stimuli other than light (Reebs & Mrosovsky, 1989; Eastman *et al*. 1995; Mrosovsky, 1996; Mistlberger & Antle, 2011; Youngstedt *et al*. 2019). Thus, PRCs provide insight into whether a cue is a zeitgeber and how it modulates the phase of a circadian oscillator, e.g. tissue‐specific clocks.

Exercise is a robust stimulus that induces significant changes in core temperature and hormonal responses, as well as tissue‐specific transcriptional and metabolic outcomes. Skeletal muscle is a large organ system and is known to be an important tissue for overall organismal health (Demontis *et al*. 2013). Exercise exerts numerous positive effects on skeletal muscle health (Cartee *et al*. 2016; Gabriel & Zierath, 2019) and proper muscle circadian function is important to maintain skeletal muscle health (Kondratov *et al*. 2006; Schroder *et al*. 2015; Chatterjee & Ma, 2016; Harfmann *et al*. 2016; Schiaffino *et al*. 2016; Dyar *et al*. 2018). To date, however, it is unclear how exercise interacts with the muscle clock and if exercise is a bona fide zeitgeber for the circadian clock in skeletal muscle. In the current investigation, we use the combination of an established mouse model of exercise and a newly designed *in vitro* cell system to demonstrate that muscle contractions as a component of exercise are indeed a zeitgeber for the circadian clock in skeletal muscle. Moreover, we also investigate how contractions modify the phase of the muscle circadian clock as well as expression of core clock genes independent of external circadian inputs. These observations will lay the foundation for further research to interrogate the connection between exercise and the muscle clock to better understand the potential for use of time‐of‐day exercise interventions for therapeutic effects.

## Methods

### Ethical approval

All animal procedures in this study were conducted in accordance with the guidelines of the University of Florida for the care and use of laboratory animals (IACUC #201809136). The use of animals for exercise protocols was in accordance with guidelines established by the US Public Health Service Policy on Humane Care and Use of Laboratory Animals.

### Treadmill exercise in PERIOD2::LUCIFERASE mice

**Figure 1:**
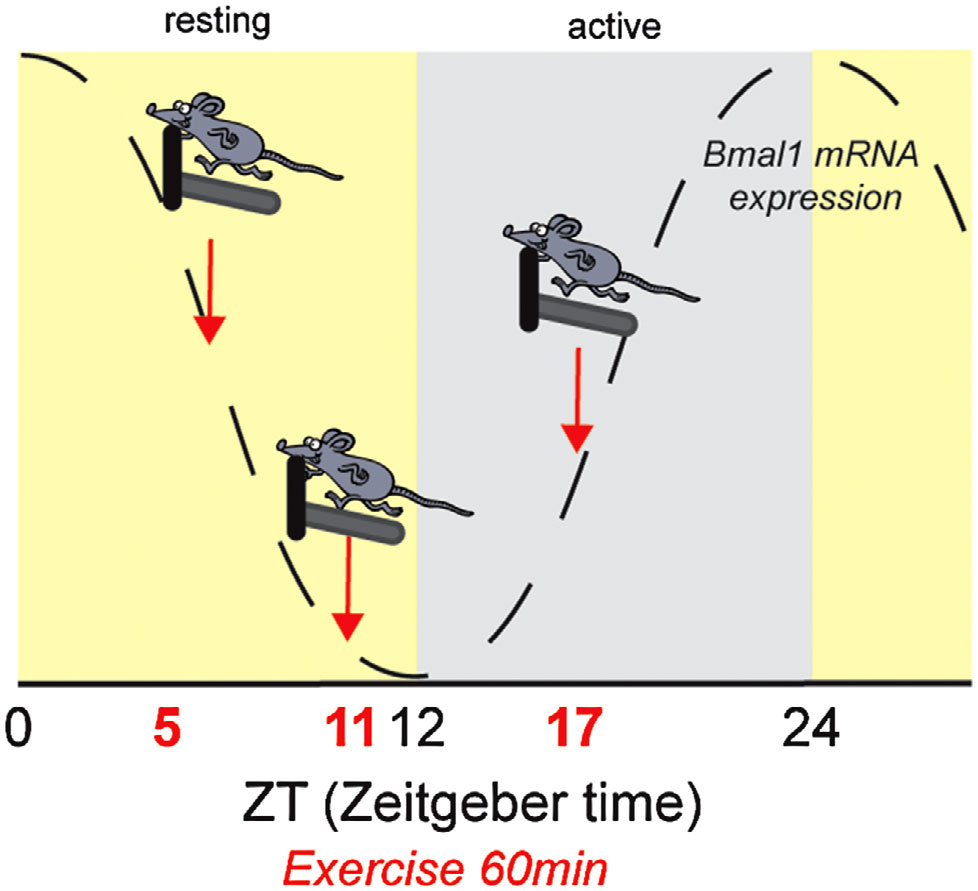
Experimental timeline for acute mouse treadmill exercise Circadian reporter (PER2::LUC) mice completed a moderate‐intensity bout of forced treadmill exercise (60 min) in the middle or end of the resting phase (ZT5 or ZT11) and in the middle of the active phase (ZT17). Timing of exercise is defined as zeitgeber time (ZT), which represents time based on the period of a zeitgeber (time cue), in this case 12 h light:12 h dark. The grey dotted line represents the relative expression of Bmal1 mRNA over time. [Color figure can be viewed at wileyonlinelibrary.com]

We used 30 female PERIOD2::LUCIFERASE (PER2::LUC) (Yoo *et al*. 2004) mice (3mo: *n* = 20 20 ± 1 g; 14mo: *n* = 10 25 ± 2 g) to test the effects of an acute bout of treadmill exercise on the circadian clock in skeletal muscle. These animals are maintained in our breeding colony and were originally a gift from Dr Joseph Takahashi. We utilized female mice, as they run more than their male counterparts (Rosenfeld 2017). Previous data from our laboratory found no sex‐specific effects of exercise training on muscle circadian phases (Wolff & Esser, 2012). Additionally, one group of mice was significantly older than the other two groups. However, this does not influence our findings, as previous data reported no impact of age on zeitgeber‐induced phase shifts in young or older (18 mo) mice (Kolker *et al*. 2004). Mice were group housed (2–5 per cage) and maintained on a 12:12 h light:dark schedule with *ad libitum* access to standard rodent chow (Envigo Teklad 2918, Indianapolis, IN, USA) and water. Mice were subjected to two days of treadmill acclimation consisting of a 10 min bout of forced treadmill exercise at the corresponding ZT with increasing speed during each bout. During the first bout, speed ramped from 15 cm/s to 20 cm/s and during the second bout, speed was ramped from 18 cm/s to 25 cm/s. Familiarizations and exercise sessions were completed at ZT5, ZT11, or ZT17 (for reference ZT0 = lights on: ZT12 = lights off: ZT17 was completed under red light) (Fig. 1). The day after the second familiarization, five PER2::LUC animals were subjected to 1 h of treadmill running at 15 m/min at 0° (EX) on a Panlab treadmill (Harvard Apparatus, Holliston, MA) while five other PER2::LUC animals were moved from the housing suite into the treadmill room and were maintained sedentary for the duration of the exercise. Food and water were removed from both groups at the onset of exercise and not returned. Following the cessation of exercise, mice were returned to their home cages for 1 h. Mice were anaesthetized using isoflurane and killed by cervical dislocation. Extensor digitorum longus (EDL) muscles were dissected from the anterior tibia of each mouse, taking care to isolate from tendon to tendon and used for real‐time bioluminescence recording.

### Cell culture, myogenic differentiation and circadian synchronization

C2C12 cells were purchased from ATCC (ATCC Cat# CRL‐3419, RRID:CVCL_UR38, LOT: 70004012). Cells were cultured in 5% CO_2_, 95% humidity at 37°C. Cells were maintained in 150 mm cell culture dishes in growth media consisting of Dulbecco's Modified Eagle's Medium (DMEM) supplemented with 10% fetal bovine serum (FBS) and 1% penicillin‐streptomycin (P/S) and 1 mm sodium pyruvate. C2C12 cells were grown to 100% confluency on 35 mm cell culture dishes that were previously coated with a gelatin hydrogel as published before with some variations to the protocol (Bettadapur *et al*. 2016; Denes *et al*. 2019). The non‐patterned hydrogel was made by dissolving 12%w/v porcine gelatin (Sigma, St. Louis, MO) in ddH_2_O by heating to 65°C and dissolving a 10% w/v solution of microbial transglutaminase (MTG) by heating to 37°C. Gelatin and MTG were mixed 10:1 and incubated for 5 min at 65°C. 120 µl of gelatin was added to the 35 mm dishes, distributed equally, and crosslinked at room temperature for 16–18 h. Gelatin‐coated plates were rehydrated with phosphate‐buffered saline (PBS) for 4 h and UV sterilized for 30 min prior to use. For cell differentiation, the medium was changed to differentiation medium (DM) consisting of DMEM supplemented with 2% horse serum (HS) and 1% P/S + 1 mm of sodium pyruvate. Cells were differentiated for 3 days before experiments were performed. For the synchronization of circadian clocks, myotubes were treated with 1 µm dexamethasone (Sigma‐Aldrich D2915) in DM for 90 min (Balsalobre *et al*. 2000). After treatment, cells were washed with PBS and received fresh DM.

### Generation of stable transfected C2C12 cells and experimental setup

**Figure 2:**
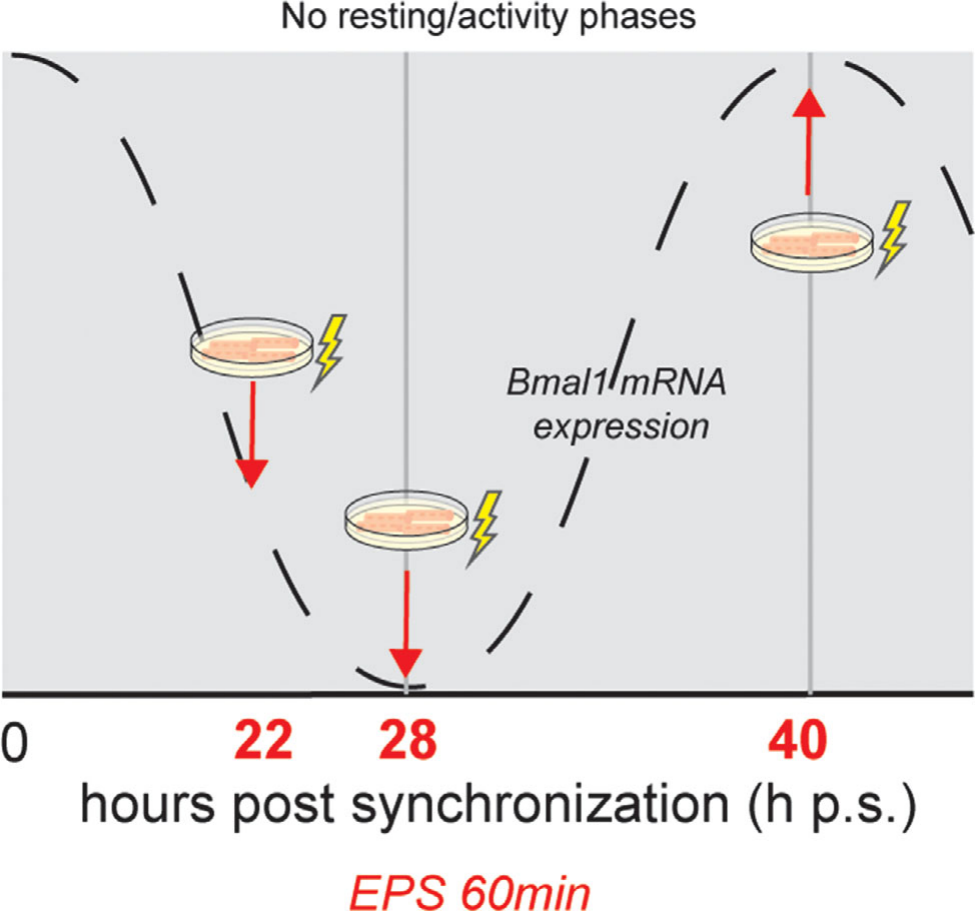
Timing of electrical pulse stimulation/contractions in myotubes in cell culture C2C12 myotubes were synchronized with dexamethasone and then subjected to electrical pulse stimulated for 1 h to induce twitch‐like contractions (lightning bolt). The timing was aligned based upon the temporal oscillation of Bmal1 mRNA content in the cells. Because C2C12 myotubes do not have rest/active phases, timing of contractions is presented as hours post‐synchronization (h p.s.) [Color figure can be viewed at wileyonlinelibrary.com]

Stable C2C12 cells were generated using non‐viral DNA transposon. The Bmal1P‐Luc reporter gene contains the wildtype mouse Bmal1 promoter sequence starting 394 bp upstream of the transcription start (TSS) and ending 154 bp downstream of the TSS and was a gift from Dr John Hogenesch (Sato *et al*. 2004). Bmal1P‐Luc was subcloned into a modified version of pminiTol2 plasmid (removal of CMV promoter and addition of pSV40neo cassette), a gift from Stephen Ekker (Addgene plasmid # 31829; http://n2t.net/addgene:31829; RRID:Addgene_31829). The transposase pCMV‐Tol2 was a gift from Stephen Ekker (Addgene plasmid # 31823; http://n2t.net/addgene:31823; RRID:Addgene_31823) (Balciunas *et al*. 2006). Transfected cells were selected for integration of the transgene over the course of one week with G418 (2 mg/ml). Clones that tested positive for transgene integration and that showed sustained bioluminescence oscillations with a period length of 23–24 h were further cultivated. For the final experiments three different clones were used to generate the data for each time point. We utilized multiple stable clones to exclude the possibility that observed results were influenced by the transgene integration into the genome. It is known that in mice Bmal1 expression in skeletal muscle peaks during transition from the dark (active) to the light (resting) phase, while the trough happens when the animals transition from the light to the dark phase. However, C2C12 cells do not receive an entraining light/dark stimulus. For this reason we decided to temporally align *in vitro* experiments with the occurrence of peaks and troughs of Bmal1 expression as seen in Figs 1 and 2.

### Electrical pulse stimulation of C2C12 myotubes

On day three, C2C12 myotubes received fresh DM 1–3 h prior to electrical pulse stimulation (EPS). EPS was carried out in 35 mm dishes using an IONOPTIX C‐Pace EP system, stimulating six dishes in the same bank. Conditions were a 25 ms pulse with 10 V at 6 Hz every 5 s for 1 h. Visual assessment of all plates revealed twitching across the field of view. Each EPS experiment included 3–5 technical replicates per treatment group (no‐stim *vs*. EPS), and each new EPS experiment was conducted using a distinct biological replicate of cells, including different clones of Bmal‐Luc stable cell lines. Myotubes were stimulated at 22 h, 28 h or 40 h post‐synchronization (Fig. 2), placed into recording medium, vacuum sealed and put into the Lumicycle for bioluminescence recording.

### RNA isolation and quantitative real‐time PCR

Three dishes (35 mm) were collected in 500 µl of Trizol each (Invitrogen 15596018) and then pooled into one tube and were treated as a single biological replicate. RNA was isolated using the RNeasy Mini Kit (Qiagen 74104) according to the manufacturer's protocol. DNAse digest was performed on column using the RNase‐Free DNase Set (Qiagen 79254) according to the manufacturer's protocol. cDNA was generated using 500 ng of total RNA using the SuperScript III First Strand Synthesis System (Thermo Fisher Scientific 18080051) according to the manufacturer's protocol. All cDNA samples were diluted 1:25 in RNAse‐free water and 4 µl were used to perform quantitative real‐time PCR (qRT‐PCR). The qRT‐PCR was carried out using the Applied Biosystems Fast SYBR Green Master Mix (Thermo Fisher Scientific 43‐856‐14) with 10 µm of each primer. Primer sequences are Bmal1for TCAAGACGACATAGGACACCT, Bmal1rev GGACATTGGCTAAAACAACAGTG; Per1for CGGATTGTCTATATTTCGGAGCA, Per1rev TGGGCAGTCGAGATGGTGTA; Per2for AAAGCTGACGCACACAAAGAA, Per2Rev ACTCCTCATTAGCCTTCACCT; Cry1for CACTGGTTCCGAAAGGGACTC, Cry1rev CTGAAGCAAAAATCGCCACCT; Cry2for CACTGGTTCCGCAAAGGACTA, Cry2rev CCACGGGTCGAGGATGTAGA; RevErbαfor TCCCAGGCTTCCGTGACCTTT, RevErbα TTGTGCGGCTCAGGAACATCA. qRT‐PCR was performed in a QuantiStudio 3 thermal cycler (Applied Biosystems, Foster City, CA). mRNA levels of target genes were normalized using *Rpl26* mRNA levels and relative quantification was calculated by using the ∆∆ct method. We used Rpl26 for normalization as it does not exhibit an oscillatory expression pattern (CircaDB) and is not responsive to EPS. To determine if the expression of a given mRNA exhibited a circadian oscillation, we utilized a single cosinor analysis (Refinetti *et al*. 2007). In order to measure the effect of EPS on circadian clock gene expression, mRNA levels of target genes were normalized using *Rpl26* mRNA (Rpl26for CGAGTCCAGCGAGAGAAGG, Rpl26rev GCAGTCTTTAATGAAAGCCGTG) levels and relative quantification was calculated by using the ∆∆ct method. Data for each independent experimental replicate are presented as the fold change from control (∆∆CT EPS/∆∆CT Con).

### Real‐time bioluminescence recordings

For real‐time bioluminescence recording, myotubes were washed with PBS after the treatment and switched to recording medium: DMEM without phenol red (Caisson DML12‐500 ml) supplemented with 2% HS, 1 mm sodium pyruvate, 1%P/S and 0.1 mm Luciferin. Tissue explants were cultured in a similar recording medium using 5% FBS in place of 2% HS. Dishes were vacuum‐sealed with a microscopy glass coverslip and placed into the Lumicycle 32 (Actimetrics, Wilmette, IL). Real‐time bioluminescence recording was performed with a sampling frequency of every 10 min for at least four consecutive days as previously described in detail by our laboratory (Wolff & Esser, 2012). We removed the first 24 h of baseline‐subtracted raw data, due to the expected measurement fluctuations within the first 24 h of recording. Trimmed data were analysed using JTK_Cycle (RRID:SCR_017962) to determine phase (lag), period length and amplitude. The Benjamini–Hochberg multiple comparison adjusted *P* value was used to assess the quality of the curve fit. For the cell culture myotube experiments we included at least three biological replicates for each time point and three to five technical replicates for each biological replicate group. No *in vitro* data were excluded. For the PER2::LUC tissue explant experiments, each EDL was considered a technical replicate for a given animal. Animals were excluded whose explanted muscles did not show any circadian oscillation (*n* = 1 at ZT5) or technical replicates (*n* = 1 at ZT11 and *n* = 2 at ZT17) were removed when noisy readings interfered with calculation of the circadian phase. One muscle ripped during preparation and could not be used (ZT5, EDL5‐2).

### Statistical analyses

Statistical analyses were completed using GraphPad Prism 8.3 (GraphPad Prism, RRID:SCR_002798). For analysis of summarized luminescence data, as well as qPCR data, we were *a priori* interested in the effect of EPS on the circadian phase (absolute change in phase) and core clock gene expression independent of time of day, and thus compared the EPS‐induced change in mRNA expression or circadian phase with untreated control samples using an unpaired Student's *t* test once we confirmed that the data were normally distributed using the Shapiro–Wilks normality tests.

For cosinor analysis a curve was fitted using a predefined period of 24 h by the least square's method. Rhythm characteristics, including 95% confidence interval, include the MESOR (middle value of the fitted cosine representing a rhythm‐adjusted mean), amplitude (half the difference between the minimum and maximum of the fitted cosine function) and the acrophase (peak of a rhythm). Significance of rhythm was determined by rejection of the zero‐amplitude hypothesis with a threshold of 95%. Pooled correlation coefficient values from each biological replicate (*n* = 3 independent experiments) were used to calculate the mean, standard deviation, and sample size for a one‐way ANOVA to examine changes in cosinor goodness of fit for the duration of the time‐course experiments. Differences between groups were considered significant at the level of *P* < 0.05. The data that support the findings of this study are available from the corresponding author upon reasonable request. All data are presented as means ± SD.

## Results

### Time‐specific effects of exercise on circadian phase in EDL

Previous data from our group found that four weeks of daily treadmill running in the rest/light phase resulted in significant shifts of the skeletal muscle clock, with no shift in the central clock in the SCN (Wolff & Esser, 2012). We sought to determine if a single 60 min bout of moderate intensity treadmill running was sufficient to alter the phase of muscle clocks. We also assessed the effects of exercise timing relative to rest/activity on muscle circadian rhythms to find out if exercise functions as a zeitgeber capable of inducing both phase advances and phase delays of the muscle clock.

**Figure 3:**
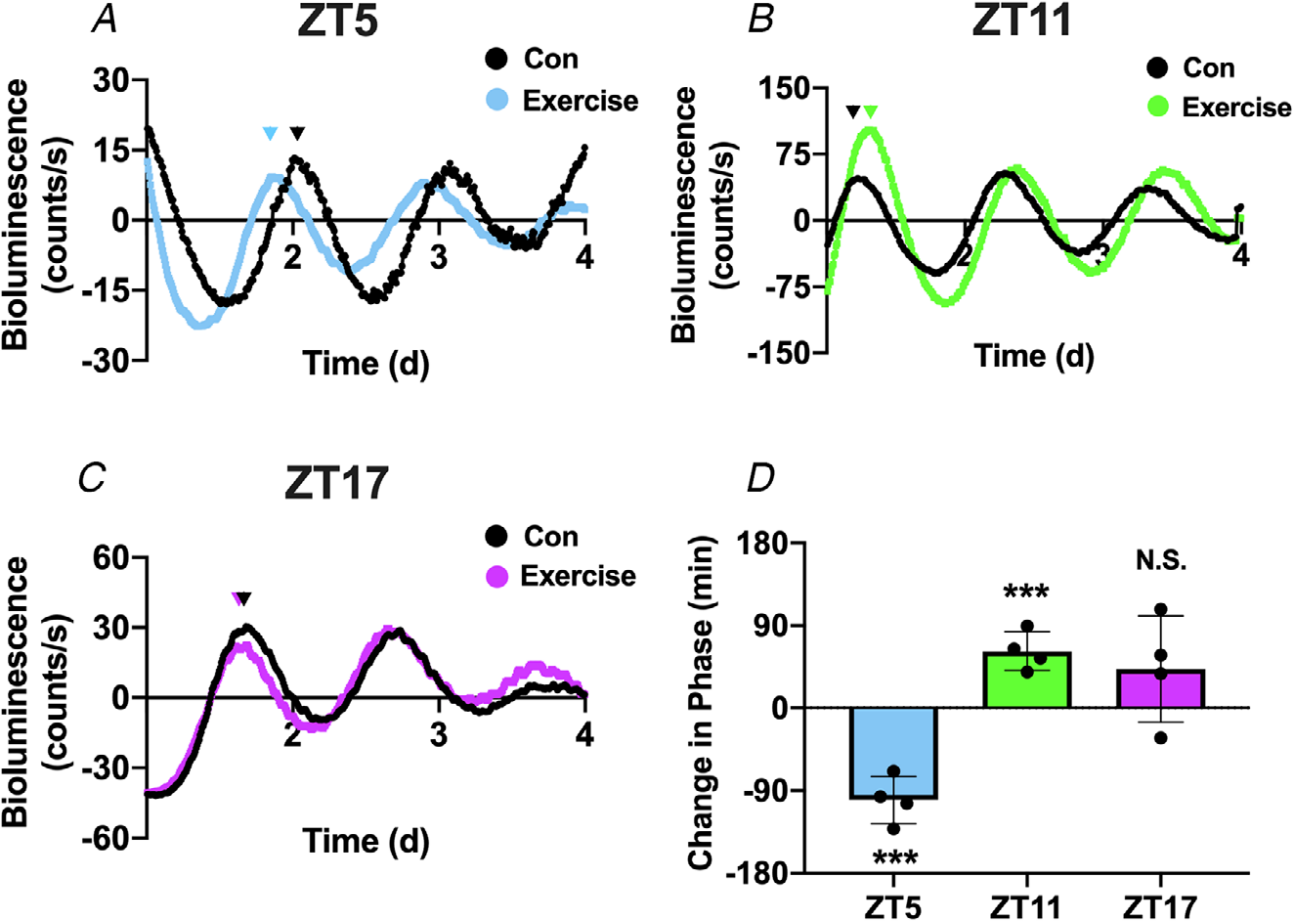
Distinct effects of exercise timing on circadian phase of skeletal muscle explants *A*, bioluminescent tracing (baseline subtracted) of PER2::LUC activity in explanted EDL muscles after 1 h of no‐exercise control (black) or treadmill exercise (blue) at ZT5. *B*, bioluminescent tracing (baseline subtracted) of PER2::LUC activity in explanted EDL muscles after 1 h of no‐exercise control (black) or treadmill exercise (green) at ZT11. *C*, bioluminescent tracing (baseline subtracted) of PER2::LUC activity in explanted EDL muscles after 1 h of no‐exercise control (black) or treadmill exercise (purple) at ZT17. *D*, quantification of the exercise‐induced change in circadian phase (minutes). A decrease in phase indicates a phase advance, and an increase in circadian phase represents a phase delay. Absolute change in phase was compared between exercise and control using an unpaired *t* test. Triangles above the bioluminescence plots represent the relative peak of circadian phase. ^***^denotes *P* < 0.001 compared with control. N.S. denotes no significant change in phase. ZT5: *P* = 0.0002; ZT11: *P* = 0.0003; and ZT17: *P* = 0.1393. [Color figure can be viewed at wileyonlinelibrary.com]

PER2::LUC mice completed a 1 h moderate intensity exercise bout at either ZT5 (middle of the light/resting phase) ZT11 (end of light/resting phase) or ZT17 (middle of the dark/active phase). EDL muscles showed PER2::LUC oscillation in control (black) and exercised (blue, green or purple) cohorts and this oscillation was maintained over several days for all three time points. We analysed changes in phase by calculating the absolute change in phase between control *vs*. exercised mice using JTK_Cycle. We found that exercise at either ZT5 or ZT11 resulted in significant changes in the phase of bioluminescence (Fig. 3*A* and *B*, respectively). Exercise at ZT5, the middle of the rest phase, induced a significant (*P* = 0.0002) phase advance of 100.1 ± 25.8 min (Fig. 3*A*) whereas exercise at ZT11, at the end of the rest phase, induced a significant (*P* = 0.0003) phase delay of 62.1 ± 21.1 min (Fig. 3B). In contrast to exercise at ZT5 and ZT11, exercise at ZT17, in the middle of the dark/active phase did not significantly shift (*P* = 0.1393) the phase of the muscle circadian clock (42.6 ± 58.1 min: Fig. 3*C*). A summary of the exercise‐induced phase changes are presented in Fig. 3*D*. Taken together, our findings demonstrate that an acute bout of treadmill running functions as a time cue for the muscle clock causing differential shifts depending on the time of exercise. We also note that one bout of treadmill exercise did not result in changes in the amplitude of period length of the EDL muscle clock (data not shown).

**Figure 4:**
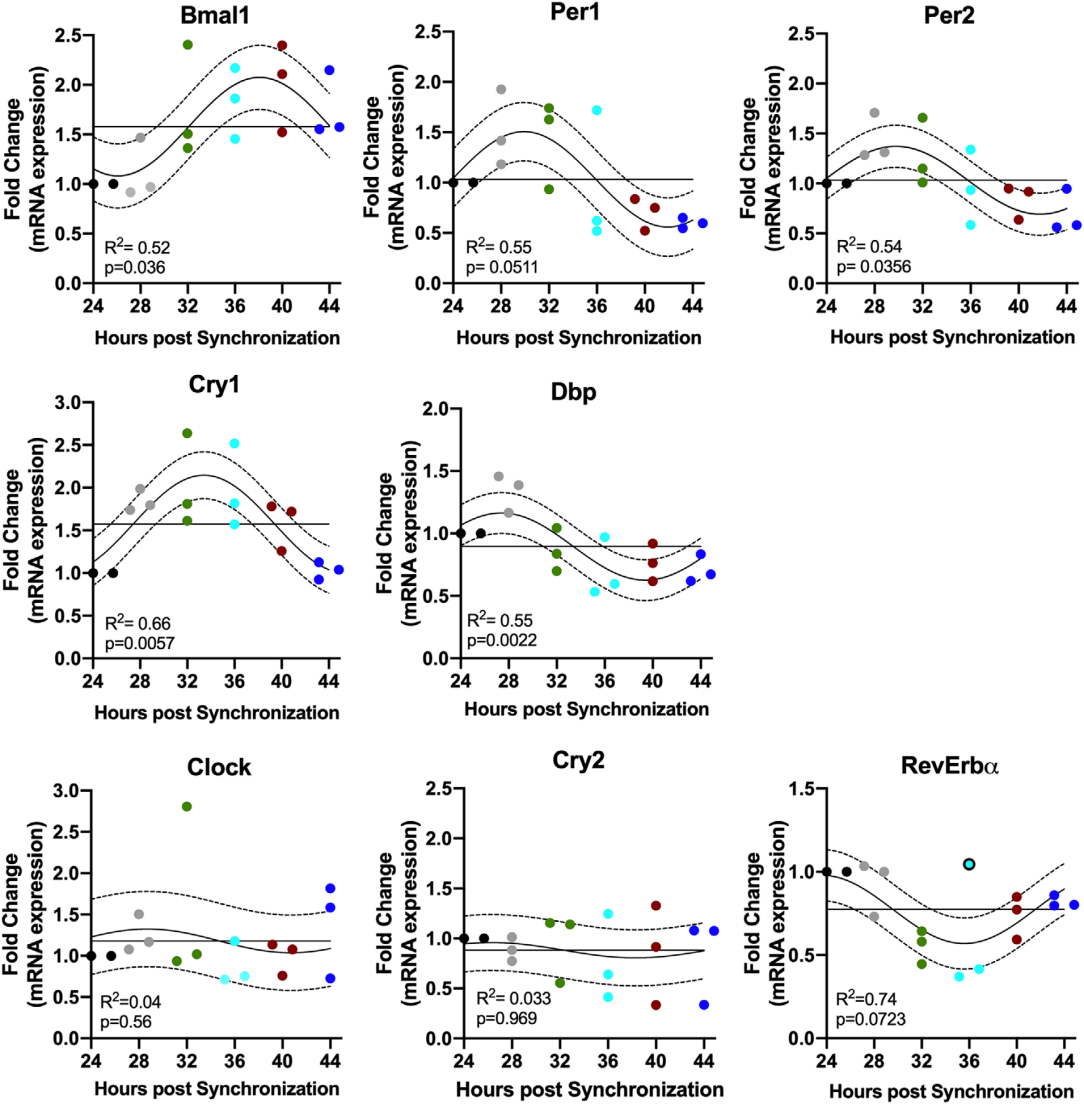
C2C12 cells exhibit an endogenous rhythm of core clock mRNA expression Twenty‐four‐hour gene expression profiles of known molecular clock‐related genes in C2C12 myotubes. Gene expression levels are represented as fold change and normalized to the initial time point (24 h). Individual values are displayed for *n* = 3 biological replicate experiments at each different time point (depicted by different colours 24 h: black; 28 h: grey; 32 h green, 36 h light blue; 40 h red; 44 h dark blue). Each biological replicate for each time point is pooled from three technical replicates (i.e. 35 mm dishes). To improve visibility of individual experimental replicates, time points where all data points were not visible were adjusted horizontally (e.g. Bmal1 28 h). The best fitting 24 h single cosine curve is shown. Dotted line represents the 95% confidence interval and the straight horizontal line the MESOR. Goodness of fit and the result of analysis of time‐of‐day variance are represented as R^2^ and *P* values, respectively. [Color figure can be viewed at wileyonlinelibrary.com]

### C2C12 myotubes exhibit an endogenous circadian rhythm

We demonstrate that a single bout of running exercise is sufficient to alter the phase of the muscle clock in a time‐specific manner. However, running is a systemic physiological stimulus that is associated with hormonal changes and core temperature changes as well as local muscle metabolism. Thus, our next set of experiments was designed to test the role of muscle contractions as a time cue for the clock. To investigate the effect of contractions on the circadian clock in skeletal muscle in an isolated system, we established an *in vitro* model, with a single cell type. C2C12 myotubes are commonly used to study various aspects of skeletal muscle physiology. In order to serve as a model for our purposes, we first ran time‐course studies to validate that the endogenous clock factors oscillate over time in C2C12 myotubes. We synchronized the phase of C2C12 myotubes with brief exposure to dexamethasone, then analysed the mRNA expression of seven core clock genes (*Bmal1, Clock, Per1, Per2, Cry1, Cry2, RevErbɑ*) as well as *Dbp*, an established clock‐output gene, every 4 h over 24 h (Fig. 3). Using cosinor analysis we determined that *Bmal1* (*P* = 0.036), *Per1* (*P* = 0.0511), *Per2* (*P* = 0.0356), *Cry1* (*P* = 0.0057) and *Dbp* (*P* = 0.002) exhibited significant circadian expression, but *Cry2* (*P* = 0.9693) and *Clock* (*P* = 0.3710) did not. Rev‐Erbɑ did not reach statistical significance (*P* = 0.0723), as one replicate from an individual experiment at the 36 h post‐synchronization time point was not in line with the others (black circled dot). We also determined that the peak of *Bmal1* mRNA expression occurred at ∼40 h post‐synchronization, while the peak of *Per1/2* expression occurred at ∼28 h, anti‐phase to *Bmal1*, as has been shown in mouse and human muscle circadian transcriptomes by McCarthy *et al*. (2007), Pizarro *et al*. (2012) and Perrin *et al*. (2018).

### One bout of electrical field stimulation changes the circadian phase in C2C12 myotubes

**Figure 5:**
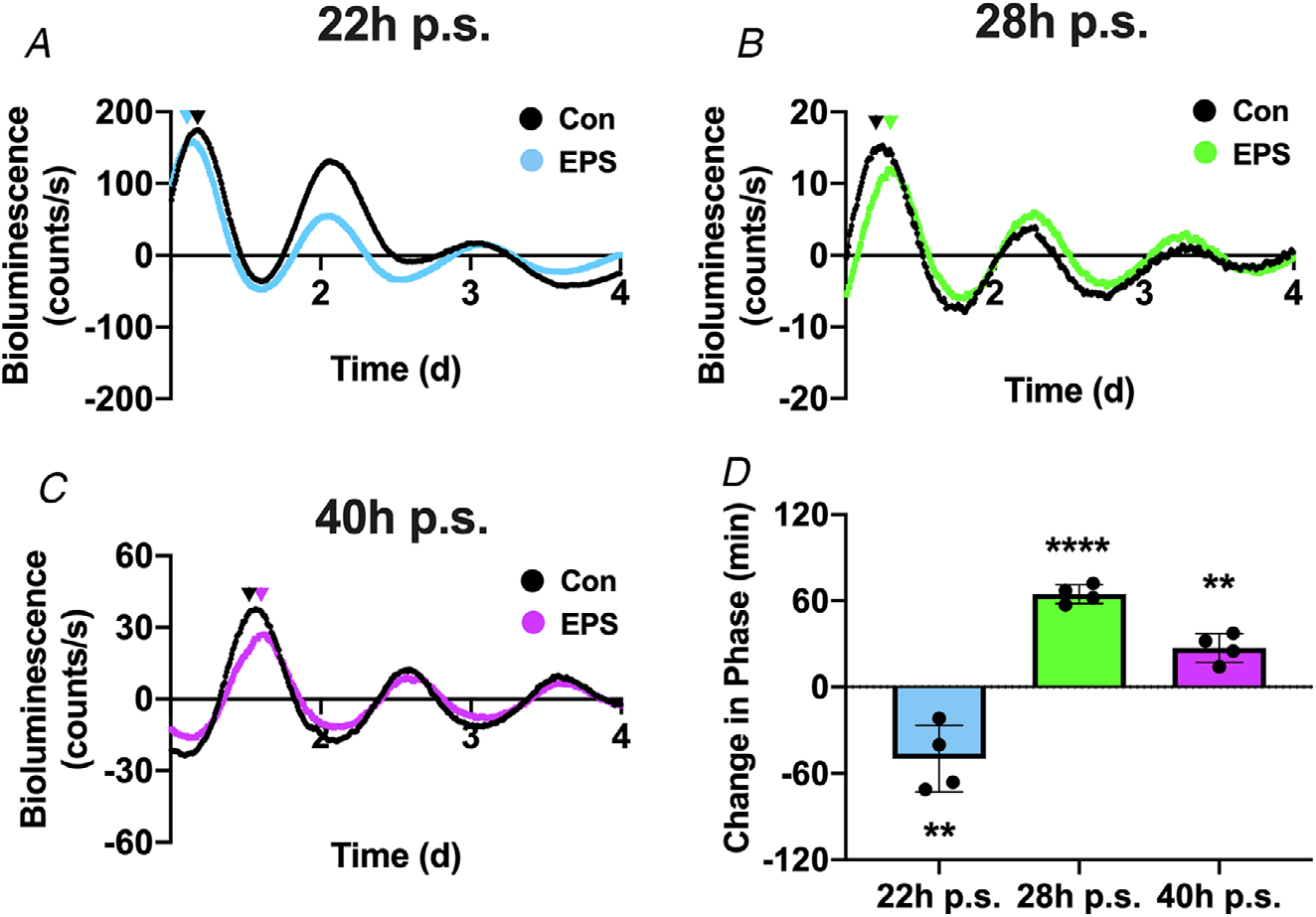
Timing of a single bout of electrical pulse stimulation‐induced contractions shift the phase of the circadian clock in C2C12 myotubes *A*, bioluminescent tracing (baseline subtracted) of Bmal1:Luc activity in cultured C2C12 myotubes after 1 h of no‐stimulation control (black) or electrical pulse stimulation (EPS)‐induced contractile activity (blue) at 22 h p.s. *B*, bioluminescent tracing (baseline subtracted) of Bmal1:Luc activity in cultured C2C12 myotubes after 1 h of no‐stimulation control (black) or EPS‐induced contractile activity (green) at 28 h p.s. *C*, bioluminescent tracing (baseline subtracted) of Bmal1:Luc activity in cultured C2C12 myotubes after 1 h of no‐stimulation control (black) or EPS‐induced contractile activity (purple) at 40 h p.s. *D*, quantification of the EPS‐induced change in circadian phase (minutes). A decrease in phase indicates a phase advance, and an increase in circadian phase represents a phase delay. Absolute change in phase was compared between EPS and control using an unpaired *t* test. Triangles above the bioluminescence plots represent the relative peak of circadian phase. ^**^denotes *P* < 0.01 compared with control and ^****^denotes *P* < 0.001 compared with control. 22 h p.s.: *P* = 0.0051; 28 h p.s.: *P *< 0.0001; and 40 h p.s.: *P* = 0.0017. [Color figure can be viewed at wileyonlinelibrary.com]

The next question we pursued was whether a single 60 min bout of contractions is sufficient to change the phase of the skeletal muscle clock (e.g. Bmal1‐Luc) in the absence of systemic factors. We generated stable C2C12 clones with a circadian clock reporter gene, Bmal1:Luc and for these experiments we used three separate clones to improve the rigour of this approach. We decided to utilize this reporter construct, where the Bmal1 promoter is driving the expression of luciferase, as this construct has been used in published work (Hughes *et al*. 2009; Hodge *et al*. 2019) and therefore is known to work reliably. Additionally, not only does the use of Bmal1:Luc *in vitro* paired with Per2::LUC *in vivo* allow us to detect the effect of exercise/contractions on both the positive and negative arm of the circadian clock, but it also allows us to be more confident that any effect of exercise/contractions on phase are truly influencing the function of the core clock mechanism, as opposed to expression of a single gene. We stimulated synchronized C2C12 myotubes (day 4 post‐differentiation) for 1 h at either 22 h following synchronization (22 h p.s. Fig. 5*A*), 28 h following synchronization (28 h p.s., Fig. 5*B*), and 40 h following synchronization (40 h p.s. Fig. 5*C*). These time points were chosen based on previously defined peak/trough expression levels of Bmal1 mRNA in the cells (see Fig. 2).

Following stimulation, the dishes were placed in the Lumicycle and phase of the Bmal1‐Luc was assessed over multiple days measuring the bioluminescence signal under static conditions (no medium change) from the Bmal1‐Luc reporter. The myotubes exhibit a clear oscillatory pattern of bioluminescence over multiple days, confirming that our cell lines expressed the integrated Bmal1:Luc transgene in proper rhythmic fashion (Fig. 5*A*–*C* left panel). We analysed the EPS‐induced changes in circadian phase as the absolute change (in minutes) from the peak of control cells. Stimulation at 22 h p.s. induced a significant (*P* = 0.0051) phase advance (49.8 ± 23.1 min Fig. 5*A*). In contrast, stimulation at 28 h p.s. led to a significant (*P *< 0.0001) phase delay of 64.6 ± 6.5 min (Fig. 5*B*), as did stimulation at 40 h p.s. (27.2 ± 10.2 min; *P* = 0.0017) (Fig. 5*C*).

### Time‐specific effects of contraction on molecular clock gene expression

**Figure 6:**
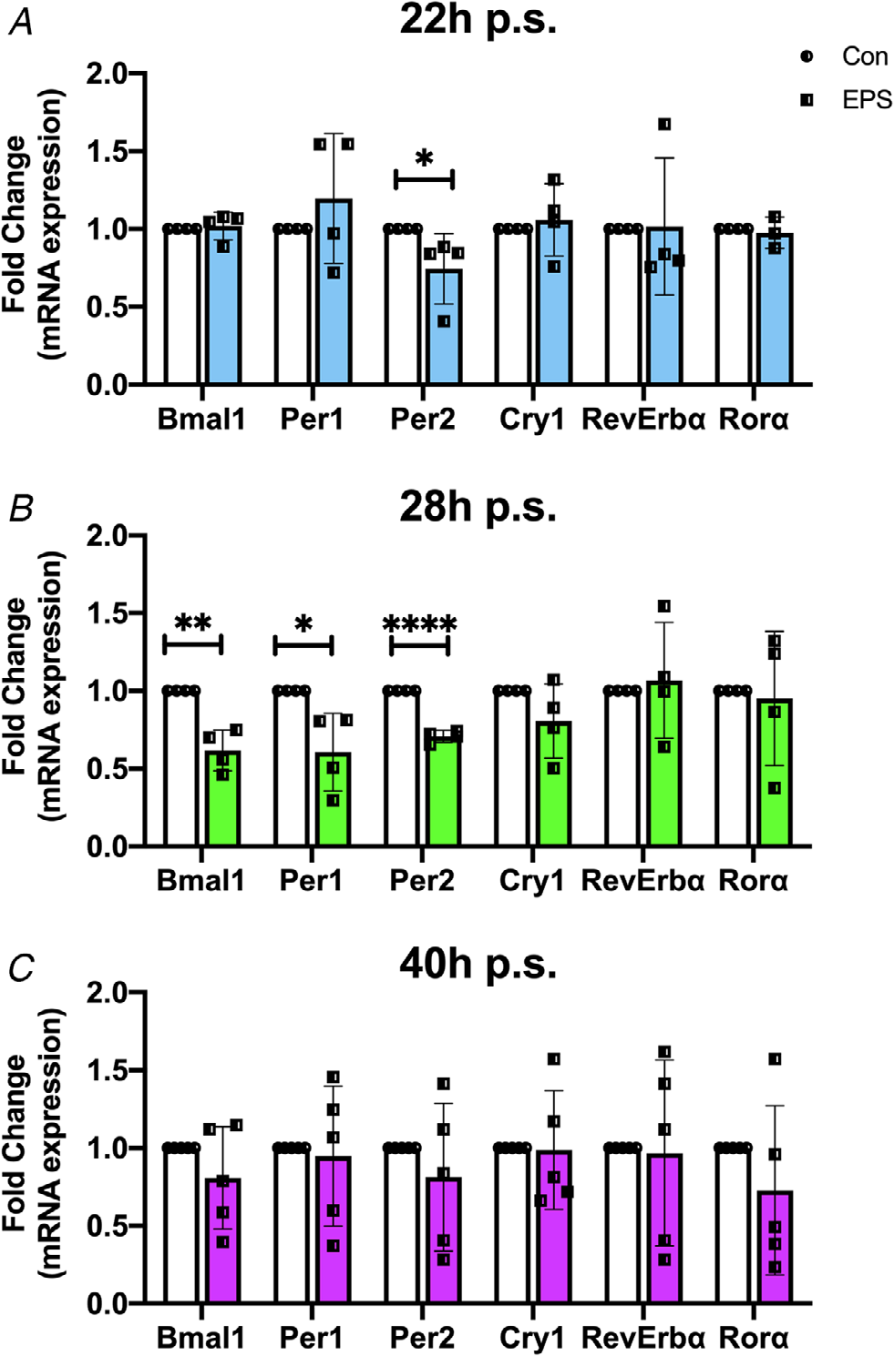
Timing of electrical pulse stimulation contractions induces distinct core clock gene expression profiles Gene expression patterns of the core clock genes Bmal1, Per1, Per2, Cry1, RevErbα, and Rorα measured immediately post‐electrical stimulation at 22 h p.s. (*A*), 28 h p.s. (*B*), or 40 h p.s. (*C*). Data are presented as fold change of control following after normalization to *Rpl26* at each time point *n* = 5 biological replicates (22 h and 28 h p.s.) *n* = 4 biological replicates (28 h p.s.) ± SD 22 h p.s.: Per2 *P* = 0.0150 (denoted by ^*^); 28 h p.s.: Bmal1 *P* = 0.0011 (denoted by ^**^), Per1 = 0.0197 (denoted by ^*^), Per2 < 0.0001 (denoted by ^****^); 40 h p.s:. *P* > 0.2 for all genes (*t* test). [Color figure can be viewed at wileyonlinelibrary.com]

We have shown that contractions induce phase shifts of different directionality depending on the time of day. This suggests that they might change the molecular clock gene programme differently depending on the time of exercise. For that reason, we investigated whether acute contractions led to changes in gene expression in the positive arm components of the circadian clock (*Bmal1/Clock*) or the negative arm of the circadian clock (*Per1/2, Cry1/2*). For this set of experiments, we stimulated C2C12 myotubes under the same conditions and collected the cells for mRNA analysis. We analysed expression of the core clock factors immediately post‐contractions. We found that contractions applied at 22 h p.s. significantly (*P* = 0.0150) reduced *Per2* mRNA expression compared with unstimulated controls by approximately 40%, but had no effect on *Per1* or *Bmal1* (Fig. 6*A*). However, when myotubes were stimulated at 28 h p.s., we found that mRNA levels for *Bmal1* (*P* = 0.0011), *Per1* (*P* = 0.0197) and *Per2* (*P* < 0.0001) were significantly decreased by around 40% compared with unstimulated controls (Fig. 6*B*) and no significant changes in any other core clock gene. Finally, unlike both other time points, myotubes stimulated at 40 h p.s. did not significantly influence the expression of any core clock genes compared with unstimulated controls (Fig. 6*C*). These results show that a single bout of acute contractions is sufficient to induce changes in the steady state mRNA levels of key components of the circadian clock in a time‐dependent manner.

## Discussion

Exercise is a well‐known stimulus that can alter skeletal muscle and organismal physiology. While emerging data have shown potent time‐of‐day specific outcomes of exercise on skeletal muscle gene expression and metabolism (Ezagouri *et al*. 2019; Gabriel & Zierath, 2019; Sato *et al*. 2019), studies of the potential role of exercise as an environmental time cue for circadian clocks have been limited. Specifically, studies have performed time‐of‐day exercise interventions and measured both behavioural as well as clock outcomes but all these studies performed repeated exercise training (Edgar & Dement, 1991; Marchant & Mistlberger, 1996; Yamanaka *et al*. 2008; Wolff & Esser, 2012). We report, for the first time, that a single 1 h bout of moderate intensity treadmill exercise is sufficient to differentially shift the phase of the circadian clock in skeletal muscle in mice. We next used an *in vitro* model system to remove the influence of systemic factors, to test the contribution of contractions to the phase of the muscle clock. We found that electrically induced contractions of muscle cells shift the phase of the circadian clock. The magnitude and direction of the time‐specific effects of contractions on phase shifts were similar in *in vivo* and *in vitro* models. Lastly, we found that the timing of contractions differentially altered the mRNA expression of core clock genes, *Per1*, *Per2* and *Bmal1*, providing molecular targets through which contractions can alter the circadian clock phase. Overall, the findings in the current investigation demonstrate that a single bout of exercise or contractions is sufficient to alter the phase of the muscle clock *in vivo* and *in vitro*. In addition, the impact of exercise on the muscle clock is dependent on the time of exercise, confirming that contractions are a bona fide muscle clock time cue.

The circadian clock has emerged as an important contributor to health and disease (Roenneberg & Merrow, 2016) and the interaction of exercise and the circadian clock has drawn attention (Wolff & Esser, 2019). Studies from as early as the eighties have shown that exercise is a time cue for circadian rhythms as interventions performed in rodents and humans shift the phase of circadian behaviour (locomotion, sleep/wake) depending on the time of exercise ( Reebs & Mrosovsky, 1989; Marchant & Mistlberger, 1996; Mrosovsky, 1996; Miyazaki *et al*. 2001; Mistlberger & Skene, 2005; Youngstedt *et al*. 2019). However, it is important to note that these early studies utilized animal behaviour as a readout for the effect of exercise on phase shifting, whereas we have directly assessed the effects of exercise on the skeletal muscle circadian clock mechanism.

Previous work from our laboratory demonstrated that repeated exercise bouts (i.e. run training) phase‐shifted the skeletal muscle clock, suggesting that exercise training could be a zeitgeber for the circadian clock in skeletal muscle. Specifically, we reported that four weeks of exercise training (∼28 exercise bouts) induced a phase advance of the muscle circadian clock (Wolff & Esser, 2012). In contrast, our current investigation is the first to report that a single 1 h bout of treadmill exercise induced a significant phase shift of the skeletal muscle circadian clock. Moreover, we performed the acute bout of exercise at three different time points over the day: the middle of the rest phase, end of the rest phase and the middle active phase. We report time‐of‐day specific effects of exercise on the muscle circadian clock. Specifically, exercise during the inactive period induced either a phase delay or phase advance, whereas exercise during the active period did not alter the circadian phase. These findings were exciting as they suggest that exercise is functioning as a true time cue for the circadian clock in skeletal muscle. In fact, since the mice were maintained in normal light:dark conditions these findings indicate that time‐of‐day exercise is sufficient to influence the phase of the muscle clock in a mouse with intact central clock and normal light cues.

Interpretation of the effects of exercise on the *in vivo* muscle circadian clock are complicated by the potential confounding influence of the central clock as well as circulating neurohumoral factors (Schibler *et al*. 2015). Thus, we sought to determine if muscle contractions, as a component of exercise, shift the phase of the muscle circadian clock under constant conditions. The capacity to test a putative time cue under constant conditions strengthens our ability to assess whether contractions act as a bona fide time cue. We found that like treadmill running, a single 1 h bout of electrical stimulation of C2C12 myotubes resulted in either a phase advance or phase delay. The phase changes were not simply an acute change to the contractions but rather the phase changes were maintained over days. These maintained changes in phase are in contrast to a local reset, where a stimulus only alters phase for a short duration. Plotting the effects of contractions on the muscle circadian phase (i.e. using the peak of Bmal1 mRNA) reveals a pattern resembling previously reported type 1 PRC, with gradual changes between advances and delays (Winfree, 1977; Glass & Winfree, 1984). While these observations indicate that contractions are likely a time cue for the skeletal muscle circadian clock, bona fide time cues must also induce direct changes in the transcriptional machinery of the circadian clock.

The effects of light on the transcriptional response of the core molecular clock in the SCN consistently upregulates Per1/2 expression, suggesting that the negative arm of the molecular clock may be targeted to alter phase (Albrecht *et al*. 1997; Shigeyoshi *et al*. 1997; Miyake *et al*. 2000). In contrast to the effect of light on the SCN, we found that contractions at 22 h p.s. and 28 h p.s. decreased Per2 and Per1/2 mRNA levels, respectively. Our findings are supported by recent data demonstrating that both acute cycling and resistance exercise downregulate Per1 at 4 h post‐exercise in humans (Dickinson *et al*. 2018). However, another investigation found that Per2 was upregulated 6 h following an acute bout of resistance exercise (Zambon *et al*. 2003). This discrepancy may be influenced by the exercise timing, as the participants with a reduction in Per1 exercised in the morning (Dickinson *et al*. 2018) and those with an increase in Per2 exercised in the early afternoon (Zambon *et al*. 2003). Although these data clearly indicate that exercise can alter the expression of the Per1 and Per2 mRNAs in skeletal muscle, our findings are novel because of our ability to link changes in clock gene expression changes with directional changes in the phase of the clock. Specifically, contractions at 22 h p.s. induced a phase advance accompanied by decreased expression of Per2 mRNA, while contractions at 28 h p.s. also lowered the expression of Per2 mRNA, yet induced a phase delay. We also identified a significant decrease in Bmal1 expression accompanied by a phase delay at 28 h p.s., which is unusual and this may reflect an additional, novel mechanism through which contractions alter the skeletal muscle circadian phase.

Exercise and electrically induced contractions induced similar effects on both direction and magnitude of circadian phase changes. In designing the *in vitro* experiments, we considered classic aspects of exercise, intensity and duration. We relied on well‐defined muscle physiology and limb muscle recruitment research from mice and rats running on a flat treadmill (Roy *et al*. 1991; de Leon *et al*. 1994). Level treadmill running at a moderate speed induces non‐tetanic, lower intensity contractions in the TA/EDL muscles. Previous data also suggest that electrically stimulated C2C12 myotubes contract, and that the contractions are synchronized with the timing of the electrical pulses (Manabe *et al*. 2012). We therefore utilized a low‐frequency stimulation paradigm *in vitro* (6 Hz) to elicit twitch and not tetanic contractions of the myotubes. Moreover, previous work has clearly defined that low‐frequency stimulation (10 Hz) paradigms *in vivo* induce an oxidative skeletal muscle phenotype, similar to endurance exercise (Delp & Pette, 1994; Patel *et al*. 1998; Nader & Esser, 2001). The animals were subjected to 1 h of forced treadmill running. We therefore kept the stimulation *in vitro* to a duration of 1 h. Finally, we acknowledge the use of distinct reporters based on experimental conditions (PER2::LUC *in vivo*; Bmal1:Luc *in vitro*). Both of these reporters are well‐established for their respective use *in vivo* and *in vitro* (Yoo *et al*. 2004; Ramanathan *et al*. 2014; Takahashi, 2017) and we contend that having a consistent effect of exercise/contraction on both Bmal1 and PER2 expression suggests a direct effect of exercise/contractions on the core circadian clock mechanism rather than just a single gene over time. Overall, we suggest that our design similarities are consistent with the concept that muscle contractions, as one component of moderate‐intensity running exercise, can lead to changes in the phase of the muscle clock.

Finally, while phase changes after contractions at 22 h and 28 h p.s. were linked to changes in mRNA expression of Per1/2 and Bmal1 this was not the case in 40 h p.s. cells. Following contractions at 40 h p.s. no core clock genes were differentially expressed compared with control. However, the contraction‐induced phase delay at 40 h p.s. was significantly less robust than the phase shift at 28 h p.s. Although it is unclear precisely what mechanism was responsible for the phase shift at 40 h p.s., previous data have suggested that post‐translational modifications also influence the molecular clock (Blau, 2008; Eng *et al*. 2017; Noguchi *et al*. 2018; Sun *et al*. 2019). Additionally, the lack of change of core clock mRNA expression, coupled with a less robust phase shift at 40 h p.s. may indicate the acute gene expression response is necessary for larger phase shifts. Future investigations using the present model system will allow for experiments that can address those questions in depth. Overall, our findings that: 1) contractions induce a shift in the phase of the muscle circadian clock in a manner that is similar to a phase 1 PRC; 2) this phase shift is maintained over time; and 3) case alterations in core clock mRNA expression levels, confirm that contractions are a bona fide time cue for the clock in skeletal muscle. In addition, the fact that contractions induce a differential outcome in terms of shifting phase and modification of molecular clock gene expression shows that there is a clear interaction between contractions and the circadian clock in skeletal muscle.

The integration of exercise physiology with circadian biology has revealed the importance of exercise timing on muscle function, metabolism and exercise performance (Tahara *et al*. 2015; Gabriel & Zierath, 2019; Wolff & Esser, 2019, 2012). The skeletal muscle circadian clock directs at least 10% of the daily transcriptional programme and contributes to daily variation in metabolic function (Hodge *et al*. 2015; Schroder *et al*. 2015; Dyar *et al*. 2018). Two recent studies showed that an acute bout of running exercise at different times of day results in significantly different skeletal muscle transcriptomic and metabolomic outcomes (Ezagouri *et al*. 2019; Sato *et al*. 2019). Our finding that acute exercise/contractions induce muscle circadian clock phase shifts is consistent with those recent studies and we suggest that some of the differential outcomes are due to the interaction between exercise and the phase of the muscle circadian clock. Additionally, the similar magnitude of exercise/contraction‐induced phase shifts *in vivo* and *in vitro* suggests that the muscle circadian mechanism is exercise‐responsive. Therefore, exercise/contraction‐induced changes in circadian phase are likely to induce profound transcriptional and metabolic remodelling, but future work is required to more carefully explore this topic. In the present study we present evidence showing that muscle contractions are a part of exercise that functions as a bona fide time cue for the skeletal muscle circadian clock. This finding could be of interest as exercise has the potential to work as a therapeutic to battle negative health outcomes linked to a lifestyle promoting circadian disruption as seen, for example, in shift workers (Morris *et al*. 2016; Jørgensen *et al*. 2017; Koshy *et al*. 2019; Zimmet *et al*. 2019). Our finding that exercise is a time cue for the skeletal muscle circadian clock in nocturnal rodents as well as in isolated muscle cells that are neither nocturnal nor diurnal, indicates that exercise could have similar effects in humans. Further, our newly developed *in vitro* system will allow for further studies aiming to elucidate the molecular mechanism that links muscle contractions to the muscle clock by providing a well‐defined and isolated system. In the future this could lead to a better understanding of how different time cues work isolated from each other but also in combination, which could lead to further improvement of therapeutic strategies combining more than one time to maximize outcome.

## Additional Information

### Competing financial interests

The authors declare no competing financial interests.

### Author contributions

D.K., C.W. and K.E. designed the experiments. D.K. and C.W. performed the experiments. D.K. and C.W analysed the data. D.K, C.W. and K.E. wrote the manuscript. All authors reviewed the manuscript and approved the final version. All authors agree to be accountable for all aspects of the work in ensuring that questions related to the accuracy or integrity of any part of the work are appropriately investigated and resolved. All persons designated as authors qualify for authorship, and all those who qualify for authorship are listed.

### Funding

This work was supported by the National Institutes of Health awards R01AR066082 and U01AG055137 to KAE.

## Supporting information


**Statistical Summary Document**

## Data Availability

All data presented in this work are available from the corresponding author upon reasonable request.
