# Supplementary material for: Time‐of‐day dependent effects of contractile activity on the phase of the skeletal muscle clock
Source: J Physiol. 2020 Jul 1;598(17):3631–44. doi: 10.1113/JP279779 (PMC7479806; doi:10.1113/JP279779)
Supplement: Supplementary file 1 — Statistical Summary Document [file TJP-598-3631-s001.docx]

**Manuscript Title:** Time of Day Dependent Effects of Contractile Activity on the Phase of the Skeletal Muscle Clock

**Authors:** Denise Kemler, Christopher A. Wolff and Karyn A. Esser

**Animal model used, if applicable: mus musculus PER2:LUC**

**Underlying hypothesis:**

Exercise at different times of day changes the circadian phase differentially in PER2::LUC mice and C2C12 myoblasts

Stimulation at different times of day changes expression of molecular clock genes differentially

**Definitions of ‘n’:**

Question 1-3: n is the number of animals in the study

Question 4 : n is the number of biological replicates

**Statistical summary table:**

| Experimental question number* | Finding/ conclusion | Experimental location/ variable  e.g. cortex vs cerebellum or genotype | Mean value  (or other summary statistic) | SD | n (value) | P** | Units | Data comparisons  e.g. WT vs KO | Statistical test | Any other variable  e.g. subjects’ age or sex | Figure/table in which data are presented | Comments  e.g. observation |
| --- | --- | --- | --- | --- | --- | --- | --- | --- | --- | --- | --- | --- |
| **Exercise-induced phase shift at ZT5?** | **Phase advance** | **EDL** | **100.2 minute phase change** | **25.8 minutes** | **5 con 4 Ex** | **0.0002** | **Minutes** | **Control vs Exercise** | **Student’s unpaired t-test** | **no** | **Figure 3A + 3D** |  |
| **Exercise-induced phase shift at ZT11** | **Phase delay** | **EDL** | **62.1 minute phase change** | **21.1 minutes** | **5 vs 5** | **0.0003** | **min** | **Control vs Exercise** | **Student’s unpaired t-test** | **no** | **Figure 3B + 3D** |  |
| Exercise-induced phase shift at ZT17 | No effect | EDL | 42.6 minute phase change | 58.1 minutes | 5 vs 4 | 0.1393 | min | Control vs Exercise | Student’s unpaired t-test | no | Figure 3C + 3D |  |
| **Does Bmal1 oscillate** | **yes** | **C2C12 myotubes** | **R^2^=0.52** | **??** | **3 per time point** | **0.036** | **AU** | **Expression over time** | **Cosinor analysis ,One way Anova** | **no** | **Figure 3** |  |
| **Does Per1 oscillate** | **yes** | **C2C12 myotubes** | **R^2^=0.55** | **??** | **3 per time point** | **0.0511** | **AU** | **Expression over time** | **Cosinor analysis ,One way Anova** | **no** | **Figure 3** |  |
| **Does Per2 oscillate** | **yes** | **C2C12 myotubes** | **R^2^=0.54** | **??** | **3 per time point** | **0.0356** | **AU** | **Expression over time** | **Cosinor analysis ,One way Anova** | **no** | **Figure 3** |  |
| **Does Cry1 oscillate** | **yes** | **C2C12 myotubes** | **R^2^=0.66** | **??** | **3 per time point** | **0.057** | **AU** | **Expression over time** | **Cosinor analysis ,One way Anova** | **no** | **Figure 3** |  |
| **Does Dbp oscillate** | **yes** | **C2C12 myotubes** | **R^2^=0.55** | **??** | **3 per time point** | **0.022** | **AU** | **Expression over time** | **Cosinor analysis ,One way Anova** | **no** | **Figure 3** |  |
| Does Clock oscillate | No | C2C12 myotubes | R^2^=0.4 | ?? | 3 per time point | 0.56 | AU | Expression over time | Cosinor analysis ,One way Anova | no | Figure 3 |  |
| Does Cry 2 oscillate | No | C2C12 myotubes | R^2^=0.033 | ?? | 3 per time point | 0.969 | AU | Expression over time | Cosinor analysis ,One way Anova | no | Figure 3 |  |
| Does RevErb oscillate | No | C2C12 myotubes | R^2^=0.74 | ?? | 3 per time point | 0.0723 | AU | Expression over time | Cosinor analysis ,One way Anova | no | Figure 3 |  |
| **EPS-induced phase shift at 22h p.s.** | **Phase advance** | **C2C12 Myotubes** | **49.8 minute phase advance** | **23.1 minutes** | **5 vs 5** | **0.0051** | **minutes** | **Control vs EPS** | **Unpaired t-test** | **no** | **Figure 5A/D** |  |
| **EPS-induced phase shift at 28hp.s.** | **Phase delay** | **C2C12 Myotubes** | **64.6 minute phase delay** | **6.5 minutes** | **4 vs 4** | **0.0001** | **minutes** | **Control vs EPS** | **Unpaired t-test** | **no** | **Figure 5B/D** |  |
| **EPS-induced phase shift at 40hp.s.** | **Phase delay** | **C2C12 Myotubes** | **27.2 minute phase delay** | **10.2 minutes** | **4 vs 4** | **0.0017** | **Fold change of h** | **Control vs EPS** | **Unpaired t-test** | **no** | **Figure 5C/D** |  |
| Effect of EPS on gene expression 22h.p.s Bmal1 | no | C2C12 myotubes | 0.99 | 0.1 | 5vs 5 | 0.815 | Fold change from Con | Control vs EPS | Unpaired t-test | no | Figure 6A |  |
| Effect of EPS on gene expression 22h.p.s Per1 | no | C2C12 myotubes | 1.21 | 0.37 | 5 vs 5 | 0.240 | Fold change from Con | Control vs EPS | Unpaired t-test | no | Figure 6A |  |
| **Effect of EPS on gene expression 22h.p.s Per2** | **yes** | **C2C12 myotubes** | **.72** | **0.20** | **5 vs 5** | **<0.0001** | **Fold change from Con** | **Control vs EPS** | **Unpaired t-test** |  |  |  |
| Effect of EPS on gene expression 22h.p.s Cry1 | no | C2C12 myotubes | 0.99 | 0.24 | 5 vs 5 | 0.985 | Fold change from Con | Control vs EPS | Unpaired t-test | no | Figure 6A |  |
| Effect of EPS on gene expression 22h.p.s RevErba | no | C2C12 myotubes | 0.96 | 0.40 | 5 vs 5 | 0.815 | Fold change from Con | Control vs EPS | Unpaired t-test | no | Figure 6A |  |
| Effect of EPS on gene expression 22h.p.s Rora | no | C2C12 myotubes | 0.93 | 0.1 | 4 vs 4 | 0.292 | Fold change from Con | Control vs EPS | Unpaired t-test | no | Figure 6A |  |
| **Effect of EPS on gene expression 28h.p.s Bmal1** | **yes** | **C2C12 myotubes** | **0.62** | **0.13** | **4 vs 4** | **0.001** | **Fold change from Con** | **Control vs EPS** | **Unpaired t-test** | **no** | **Figure 6B** |  |
| **Effect of EPS on gene expression 28h.p.s Per1** | **yes** | **C2C12 myotubes** | **0.61** | **0.25** | **4 vs 4** | **0.0197** | **Fold change from Con** | **Control vs EPS** | **Unpaired t-test** | **no** | **Figure 6B** |  |
| **Effect of EPS on gene expression 28h.p.s Per2** | **yes** | **C2C12 myotubes** | **0.71** | **0.038** | **4 vs 4** | **<0.0001** | **Fold change from Con** | **Control vs EPS** | **Unpaired t-test** | **no** | **Figure 6B** |  |
| Effect of EPS on gene expression 28h.p.s Cry1 | no | C2C12 myotubes | 0.81 | 0.24 | 4 vs 4 | 0.156 | Fold change from Con | Control vs EPS | Unpaired t-test | no | Figure 6B |  |
| Effect of EPS on gene expression 28h.p.s RevErba | no | C2C12 myotubes | 0.96 | 0.40 | 4 vs 4 | 0.729 | Fold change from Con | Control vs EPS | Unpaired t-test | no | Figure 6B |  |
| Effect of EPS on gene expression 28h.p.s Rora | no | C2C12 myotubes | 0.95 | 0.43 | 4 vs 4 | 0.826 | Fold change from Con | Control vs EPS | Unpaired t-test | no | Figure 6B |  |
| Effect of EPS on gene expression 40h.p.s Bmal1 | no | C2C12 myotubes | 0.81 | 0.33 | 4 vs 4 | 0.228 | Fold change from Con | Control vs EPS | Unpaired t-test | no | Figure 6C |  |
| Effect of EPS on gene expression 40h.p.s Per1 | no | C2C12 myotubes | 0.95 | 0.45 | 5 vs 5 | 0.802 | Fold change from Con | Control vs EPS | Unpaired t-test | no | Figure 6C |  |
| Effect of EPS on gene expression 40h.p.s Per2 | no | C2C12 myotubes | 0.81 | 0.48 | 5 vs 5 | 0.402 | Fold change from Con | Control vs EPS | Unpaired t-test | no | Figure 6C |  |
| Effect of EPS on gene expression 40h.p.s Cry1 | no | C2C12 myotubes | 0.99 | 0.39 | 5 vs 5 | 0.949 | Fold change from Con | Control vs EPS | Unpaired t-test | no | Figure 6C |  |
| Effect of EPS on gene expression 40h.p.s RevErba | no | C2C12 myotubes | 0.97 | 0.60 | 5 vs 5 | 0.908 | Fold change from Con | Control vs EPS | Unpaired t-test | no | Figure 6C |  |
| Effect of EPS on gene expression 40h.p.s Rora | no | C2C12 myotubes | 0.73 | 0.54 | 5 vs 5 | 0.296 | Fold change from Con | Control vs EPS | Unpaired t-test | no | Figure 6C |  |

*You may use multiple lines for the same question to indicate multiple comparisons

** Authors may wish to make the text bold where p is considered significant against a stated confidence limit
